# Physical activity and cognitive function: moment-to-moment and day-to-day associations

**DOI:** 10.1186/s12966-023-01536-9

**Published:** 2023-11-22

**Authors:** Tiia Kekäläinen, Martina Luchetti, Antonio Terracciano, Alyssa A. Gamaldo, Jacqueline Mogle, Hephzibah H. Lovett, Justin Brown, Timo Rantalainen, Martin J. Sliwinski, Angelina R. Sutin

**Affiliations:** 1 Gerontology Research Center and Faculty of Sport and Health Sciences, University of Jyväskylä, Jyväskylä, Finland; 2 Department of Behavioral Sciences and Social Medicine, College of Medicine, Florida State University, Tallahassee, FL USA; 3 Department of Geriatrics, College of Medicine, Florida State University, Tallahassee, FL USA; 4 Human Development and Family Studies, The Pennsylvania State University, Pennsylvania, PA USA; 5 Department of Psychology, Clemson University, Clemson, SC USA

**Keywords:** Cognition, Accelerometry, Ambulatory assessment, Naturalistic setting, Ecological momentary assessment

## Abstract

**Background:**

The beneficial effect of acute physical exercise on cognitive performance has been studied in laboratory settings and in long-term longitudinal studies. Less is known about these associations in everyday environment and on a momentary timeframe. This study investigated momentary and daily associations between physical activity and cognitive functioning in the context of everyday life.

**Methods:**

Middle-aged adults (*n* = 291, aged 40–70) were asked to wear accelerometers and complete ecological momentary assessments for eight consecutive days. Processing speed and visual memory were assessed three times per day and self-rated evaluations of daily cognition (memory, thinking, and sharpness of mind) were collected each night. The number of minutes spent above the active threshold (active time) and the maximum vector magnitude counts (the highest intensity obtained) before each cognitive test and at a daily level were used as predictors of momentary cognitive performance and nightly subjective cognition. Analyses were done with multilevel linear models. The models were adjusted for temporal and contextual factors, age, sex, education, and race/ethnicity.

**Results:**

When participants had a more active time or higher intensity than their average level within the 20 or 60 minutes prior to the cognitive test, they performed better on the processing speed task. On days when participants had more active time than their average day, they rated their memory in the evening better. Physical activity was not associated with visual memory or self-rated thinking and sharpness of mind.

**Conclusions:**

This study provides novel evidence that outside of laboratory settings, even small increases in physical activity boost daily processing speed abilities and self-rated memory. The finding of temporary beneficial effects is consistent with long-term longitudinal research on the cognitive benefits of physical activity.

**Supplementary Information:**

The online version contains supplementary material available at 10.1186/s12966-023-01536-9.

## Introduction

There is a consistent positive association between physical activity and cognitive functioning: More physically active people have better cognitive functioning and less cognitive decline with age than less active people [1, 2]. In addition, both structured long-term physical activity interventions [3] and acute exercise sessions [4–6] have positive effects on cognitive functioning; although not all interventions find this effect (e.g., [7]) and the effect may vary depending on the cognitive task [4, 5]. Behavioral, neurophysiological, and neurochemical mechanisms may explain the short- and long-term effects of physical activity on cognition [6, 8]. Some mechanisms, such as increased blood flow in the brain and stimulation of neurotransmitters, are activated immediately during a single bout of physical activity and diminish within hours, while other mechanisms, such as stress relief and improvement in positive mood, may last longer [6, 8]. To date, however, most of what is known about the relation between physical activity and cognitive function comes from laboratory settings or intervention-based studies. Less is known about the benefits of physical activity on cognitive functioning in the real-world, everyday environment.

Both physical activity and cognitive performance are dynamic and fluctuate in daily life, even across the course of a single day. Although accelerometers have been available to capture such fluctuations in physical activity, only recent advances in technology have made it possible to embed cognitive tasks within ecological momentary assessments (EMA) to capture cognitive functioning in real-life contexts [9, 10]. The combination of EMA with accelerometer-assessed physical activity provides a unique and robust opportunity to combine these momentary assessments with detailed time-stamped information on physical activity [11]. Analyses of within-person (intra-individual) associations reveal more nuanced dynamic associations between variables in daily life compared to between-person analyses [12].

Studies applying the EMA concept to investigate within-person effects of physical activity in daily life have shown that when individuals are more physically active than they typically are, they report better mood, more energetic feelings, and lower perceived stress compared to their own average level [11, 13–15]. The within-person association between physical activity and cognitive performance is less studied. Most studies on within-person associations have found physical activity to be related to executive function. A study among adults aged 50–74 (*n* = 90) found that across 2 weeks, days with greater accelerometer-based physical activity were associated with faster executive function but not verbal learning or recall [16]. The same participants also had lower executive function when they reported currently doing passive activities [17]. A study among adults aged 60+ (*n* = 51) found no statistically significant within-person association between daily accelerometer-based physical activity and cognitive function assessed on the same day, but previous day physical activity explained the within-person variance for processing speed [18]. In a study on perceived cognitive ability, college students (*n* = 128) reported greater perceived cognitive ability on days when they were more physically active than usual [19]. In all these studies, each cognitive test was performed once a day either by smartphones with randomized prompts [16, 17], self-selected time on a web page [19], or at a local day center [18]. Thus, the momentary associations between physical activity and cognition were limited by the single cognitive assessment per day and the analyses focused mainly on day-to-day, not moment-to-moment, variation.

Laboratory-based studies suggest that exercise sessions should be at least 20 minutes to have cognitive benefits and that the most significant improvement in cognitive performance is observed approximately 15 minutes after exercise, depending on the intensity [4, 5]. While light intensity exercise can provide immediate benefits, more intense exercise may be necessary to achieve delayed effects [5]. However, the temporal associations in daily life context may be different. Studies examining the momentary associations between physical activity and mood and affective states suggest that engaging in physical activity of any intensity, or even replacing sedentary time with standing, is associated with better mood and feelings of energy on a within-person level [15, 20, 21]. These associations appear to be similar in both 15- and 30-minute epochs [15, 20]. However, the beneficial effect of physical activity on mood in the previous 60 minutes decreases over a three-hour time window [21]. Similar temporal associations may exist between physical activity and cognitive performance.

The purpose of the present study was to assess the momentary and daily association between physical activity assessed by accelerometers and cognitive function assessed with momentary performance three times a day and subjective evaluations nightly among middle-aged adults. We posed the following research questions:

- RQ1. Does physical activity during the preceding 20 or 60 minutes predict cognitive performance? (within-person, momentary-level).
- RQ2. Does daily physical activity predict self-rated cognition of the day? (within-person, day-level).

We hypothesized a similar positive association between physical activity and subsequent cognitive performance in daily life, as previously found in laboratory settings [4, 5]. The selected time frames of 20 and 60 minutes were based on previous findings that the effects of exercise on cognition subside following over 20 minutes delay [5] and that engaging in physical activity is associated with a better mood in a one-hour time window but not in a three-hour window [21]. We expected to replicate the finding of better perceived cognition on days with more physical activity than usual observed for younger samples [19] with our sample of middle-aged adults.

## Methods

### Participants and the procedure

The data were from adults in the United States who participated in the Couples Healthy Aging Project (CHAP) (*n* = 308). Participants were recruited through social media advertisements, community events, and snowball sampling. The inclusion criteria were 1) both members of the couple were aged 40 to 70 years, 2) in a committed relationship for at least 1 year and cohabitating, and 3) both members of the couple were free of severe cognitive impairment (The modified Telephone Interview for Cognitive Status score > 6 [22, 23]) and willing to enroll in the study. All procedures and materials were approved by the Institutional Review Board of the Florida State University (ID: STUDY00000472).

**Fig. 1:**
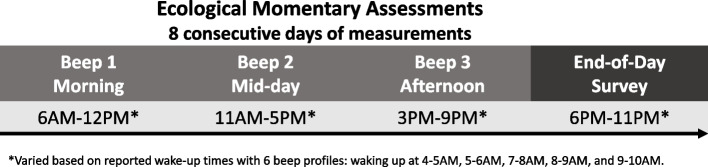
The schedule for Ecological Momentary Assessments

Eligible and interested participants were invited to an online meeting in which, after the informed consent process, participants completed a battery of cognitive tests. A study-provided smartphone and accelerometer were delivered to participants, and they were asked to wear the accelerometer and complete the ambulatory assessments for eight consecutive days. The smartphone alerted participants at three semi-random times to complete a brief assessment, including a battery of cognitive tests and a survey about their day each night (Fig. 1). The beep windows varied based on reported wake-up times with six possible beep profiles. The morning window varied between 6 am to 12 pm (average 9:14 am), the mid-day window between 11 am and 5 pm (average 2:29 pm), and the afternoon window between 3 pm and 9 pm (average 6:20 pm). The end-of-day survey was beeped between 6 pm and 11 pm (average 9:09 pm). Participants were allowed self-initiation to make up for missed notifications and forced interruptions during a survey. The median time lag between a beep and the start of the response was 0.27 minutes (range 0.02–289.02).

Of the 308 participants recruited, 98% (*n* = 303) had valid data from the EMA portion of the study (*n* = 5 data were lost due to technical problems with the phone) and 96% (*n* = 296) wore the accelerometer. The present study includes participants who had information on both EMA and physical activity from at least 1 day (*n* = 291). The analytic sample did not differ from the rest of the recruited participants in terms of sociodemographic factors, health status, or cognitive functioning (Additional File 1).

The data were collected between February 2020 and October 2021. Because of the onset of the COVID-19 pandemic, data collection was temporally suspended and resumed in June 2020. Assessments and procedures were modified to allow participants to complete all study components remotely.

### Measures

#### Ambulatory cognition

Participants completed two cognitive tests validated for ambulatory assessment: the Symbol Search Task (SST) assessed processing speed and the Dot Memory test (DMT) assessed visual memory [24]. These tasks assess two fundamental and distinct cognitive functions [24]. In the SST, participants match symbol pairs as quickly as possible. Each test session comprised 12 trials and the mean response time of correct trials for each session was calculated. In the DMT, participants saw three dots in a 5 × 5 checkerboard for 3 seconds, and after an 8-second filler task, they were asked to indicate the location of the red dots. The average distance between the correct and the indicated location of the dots across each trial (two trials for each test session) was used in the analyses. In both cognitive tests, a larger value (slower reaction time, more errors) indicated worse performance.

#### Self-rated cognition

In each night survey, participants rated whether their mind was as sharp, their memory as good, and their thinking as fast as usual today [25, 26]. The response scale was from 0 to 100, with a higher value indicating better cognition.

#### Physical activity

ActiGraph (ActiGraph Corp., Pensacola, FL) wrist-worn tri-axial accelerometers were used to measure physical activity for 8 days. Participants were instructed to wear the device on their wrists for 24 hours per day for the same eight consecutive days that they took part in the ambulatory assessment. They were instructed to take the device off only for showers and water-based activities.

ActiGraph data were analyzed using the ActiLife (ActiGraph Manufacturing Technology Inc., FL) Software. A non-wear time was defined as 90 minutes of continuous zero vector magnitude counts (VMC) [27, 28]. Days with at least 10 hours of wear time were included in the analyses [29]. Wear time was full 24 hours for 83% of days. The data were analyzed in 60-s epochs and divided into sedentary and active time based on the cut-points of < 2303 VMC per minute (cpm) for the dominant wrist and < 1853 cpm for the non-dominant wrist [30].

Using the time stamps from the smartphones and accelerometers, the timing of EMA prompts was synchronized with the accelerometer data. Custom-written MATLAB (version R2019b, The MathWorks Inc., Natick MA, USA) scripts were used to extract the specific period (20 and 60 minutes) before each EMA assessment and the time between the last activity minute and the EMA assessment. Active time (minutes) and maximum counts were used as indicators of physical activity in the present study. Active time is an indicator of time spent other than sedentary activities, whereas maximum counts indicate the maximum intensity of physical activity reached. VMC and steps were reported for descriptive purposes.

#### Demographics

Age in years, sex (0 = male, 1 = female), race/ethnicity (0 = white, 1 = person of color), and education in (years) were asked in the main interview.

### Statistical analyses

Statistical analyses were performed with IBM SPSS Statistics Version 28.0.1.1. (IBM Corp. in Armonk, NY) and R Version 4.2.1. (R Foundation for Statistical Computing, Vienna, Austria). Data were prepared for analysis and check for quality following recommendations [31]. Means, standard deviations, frequencies, and correlations were used for descriptive purposes. Within-person correlations were calculated using the R package *misty* [32]*.*

The data were analyzed with multilevel models to account for the hierarchical structure of the data (days and moments nested within individuals). Level 1 repeated assessments of physical activity were person-mean centered (i.e., each momentary value of physical activity minus the mean of physical activity across assessments; 0 represents the within-person mean for each participant) to estimate when participants were more or less physically active than their average. Level 2 between-person variables were grand-mean centered (i.e., person overall mean minus grand mean; 0 represents the mean for all participants). A third level (cognitive assessments nested within participants within couples) was also tested to account for participants recruited in pairs. The variance explained by between-couples was not significant after accounting for between-person variables (age, sex, education, race/ethnicity) and thus, the results for the two-level analyses are reported.

For each cognitive outcome, a null model without any predictors was estimated to separate the within- and between-person variance using intraclass correlation coefficients (ICCs). Next, between-person and within-person predictors of interest were included in the model. The models were adjusted for between-person covariates of age (grand-mean centered), education (grand-mean centered), sex, and race/ethnicity. Temporal covariates included in the models were weekday (weekend = 0, weekday = 1) to account for weekly rhythm, day in the study (range 1–8) to account for practice effects, and time window (1 = morning, 2 = mid-day, 3 = afternoon) to account for time-dependent variation within days. Contextual covariates were location (0 = home, 1 = other) and company (0 = alone, 1 = presence of other person) at the time of the assessment to account for possible distractions. For day-level analysis, only the first two temporal covariates were included. Additionally, the accelerometer wear time for each day was included in the models assessing the associations between active time and self-rated cognition. Models were run separately for active time and maximum counts, and physical activity extracted 20 or 60 minutes before each EMA session. The Variance Components (VC) random covariance matrix was used in the analyses. The data were analyzed with restricted maximum likelihood (REML) estimation using all available data to estimate the model parameters (Additional File 1).

Three sets of additional analyses were conducted and presented in Additional File 3. To account for between-person differences, the grand-mean centered physical activity (i.e., person overall mean minus grand mean; 0 represents the mean for all participants) was included in the models. To account for the time lag between the latest physically active minute and the EMA assessment, a supplementary analysis was performed for cases having at least one physically active minute in the 20- (78.6% of cases) or 60-minute (94.2% of cases) epoch preceding the EMA assessment. To account for the response delay, a supplementary analysis was performed by excluding cases that took more than 15 minutes to respond to the prompt [33].

## Results

### Descriptive statistics

The participants’ characteristics are shown in Table 1 for participants (*N* = 291) with at least 1 day of accelerometer data from the EMA days. Of these participants, all had information on self-rated cognition from at least 2 days (93% of participants had data from 6 or more days) and all except one on cognitive tests from at least seven sessions (82% of participants completed 20 or more sessions). The bi-variate correlations between study variables are in Supplementary Table S1 (Additional File 3).

**Table 1:** Descriptive statistics for the sample (*n* = 291)

	M/N	SD/%
Sex		
Women, %	159	54.6
Men, %	132	45.4
Race/ethnicity		
White, %	215	73.9
Other, %	76	26.1
Age, years	51.9	7.4
Education, years	16.6	3.3
Complete EMA night sessions	7.4	1.2
Complete EMA day sessions	21.7	2.9
**Day level**		
Self-rated sharpness of mind	71.7	16.5
Self-rated memory	71.9	16.2
Self-rated thinking	72.0	16.3
Wear time per day, hours	22.9	0.9
Active time per day, mins	354.7	104.3
Maximum counts daily average, 10^3^	12.7	3.2
VMC per day, 10^3^	1980.5	614.9
Steps per day	4077.9	2312.6
**EMA level**		
SST, ms	1621.1	443.9
DMT, Euclidean distance	1.5	0.7
Active time 60min^a^, mins	24.5	15.5
Active time 20min^b^, mins	8.4	6.3
Maximum counts 60min^a^, 10^3^	13.7	9.2
Maximum counts 20min^b^, 10^3^	4.7	3.5

VMC Vector Magnitude Counts, SST The Symbol Search Task, mean response time, DMT The Dot Memory test, error mean (Euclidean distance), ^a^data from 60 mins period before each EMA session, ^b^data from 20 mins period before each EMA session

Across all participants over the 8 days, there were 6221 EMA assessments of processing speed and corresponding physical activity, 6045 EMA assessments of visual memory and corresponding physical activity, and 2012 nightly assessments of self-rated cognition and physical activity on the same day.

### Multilevel models

The ICCs suggest that 65% of the total variance in processing speed was between participants and 35% was within people; corresponding values for visual memory were 27 and 73%, respectively. For the self-reported outcomes, 59% of the variance in memory and 58% of the variance in thinking and sharpness of mind were attributable to between-person and 41 and 42% within-person, respectively.

The results from multilevel models are shown in Tables 2, 3 and 4. At the momentary-level (RQ1), participants performed better on the processing speed task when they were more physically active than their usual (Table 2). The same association was apparent in all four models including either maximum counts or activity minutes as a predictor and either 20 or 60 minutes before the cognitive assessment. For example, each one-minute increase in physical activity during the 20-min period before cognitive assessments was associated with 3.11 milliseconds faster processing speed (B = -3.11, SE = 0.70, *p* < .0.001) and every 1000 counts increase in maximum physical activity intensity during the 20-min period before cognitive assessments was associated with 0.5 milliseconds faster processing speed (B = -0.50, SE = 0.13, p < .0.001).

**Table 2:** Associations between physical activity and processing speed analyzed by multilevel modelling (*n* = 6221)

	Model: Max Counts 60 min				Model: Max Counts 20 min				Model: Active time 60 min				Model: Active time 20 min			
	B	SE	p		B	SE	p		B	SE	p		B	SE	p	
**Fixed effects**																
Intercept	1849.93	54.71	<.001		1843.76	54.61	<.001		1823.62	54.48	<.001		1825.36	54.47	<.001	
EMA day	−27.49	1.78	<.001		−27.44	1.78	<.001		−27.52	1.78	<.001		−27.46	1.78	<.001	
EMA session number	4.28	4.87	.380		4.00	4.87	.412		5.10	4.88	.296		4.64	4.87	.341	
Weekend	−8.78	9.43	.352		−9.35	9.43	.321		−8.70	9.43	.356		−9.04	9.43	.338	
Company	−23.27	9.05	.010		−23.86	9.06	.008		−24.36	9.06	.007		−24.70	9.07	.006	
Location	−21.83	9.30	.019		−24.31	9.30	.009		−21.01	9.31	.024		−23.36	9.29	.012	
Age^a^	23.48	3.22	<.001		23.56	3.22	<.001		23.66	3.22	<.001		23.65	3.22	<.001	
Education^a^	−1.67	7.06	.813		−1.61	7.06	.820		−1.57	7.07	.824		−1.57	7.07	.824	
Sex	123.86	47.20	.009		124.58	47.23	.009		127.00	47.27	.008		127.09	47.27	.008	
Race/ethnicity	176.92	54.05	.001		176.81	54.09	.001		178.12	54.14	.001		178.02	54.14	.001	
Physical activity^b^	−.20	.05	<.001		−.50	.13	<.001		−1.26	.30	<.001		−3.11	.70	<.001	
**Variance components**	**Estimate**	**SE**	**Wald Z**	**p**	**Estimate**	**SE**	**Wald Z**	**p**	**Estimate**	**SE**	**Wald Z**	**p**	**Estimate**	**SE**	**Wald Z**	**p**
Residual	95,534.17	1764.47	54.14	<.001	95,531.31	1764.41	54.14	<.001	9549.35	1763.66	54.14	<.001	99,671.15	1841.34	54.13	<.001
Random intercept	149,485.96	12,793.43	11.68	<.001	149,708.39	12,811.99	11.69	<.001	149,977.38	12,834.40	11.69	<.001	15,128.72	12,959.85	11.67	<.001

^a^Grand-mean centered, ^b^Person-mean centered, Reference categories female (sex), weekday (weekend), white (Race/ethnicity), home (location), with others (company)

**Table 3:** Associations between physical activity and visual memory analyzed by multilevel modelling (*n* = 6045)

	Model: Max Counts 60 min				Model: Max Counts 20 min				Model: Active time 60 min				Model: Active time 20 min			
	B	SE	p		B	SE	p		B	SE	p		B	SE	p	
**Fixed effects**																
Intercept	1.94	.11	<.001		1.95	.11	<.001		1.94	.11	<.001		1.94	.11	<.001	
EMA day	−.05	.01	<.001		−.05	.01	<.001		−.05	.01	<.001		−.05	.01	<.001	
EMA session	.08	.02	<.001		.08	.02	<.001		.08	.02	<.001		.08	.02	<.001	
Weekend	.00	.04	.943		.00	.04	.951		.00	.04	.950		.00	.04	.965	
Company	−.05	.03	.129		−.05	.03	.119		−.05	.03	.124		−.05	.03	.107	
Location	.03	.03	.422		.03	.03	.431		.03	.03	.413		.03	.03	.423	
Age^a^	.03	.01	<.001		.03	.01	<.001		.03	.01	<.001		.03	.01	<.001	
Education^a^	−.03	.01	.015		−.03	.01	.015		−.03	.01	.015		−.03	.01	.015	
Sex	−.35	.08	<.001		−.35	.08	<.001		−.35	.08	<.001		−.35	.08	<.001	
Race/ethnicity	.33	.09	<.001		.33	.09	<.001		.33	.09	<.001		.33	.09	<.001	
Physical activity^b^	.00	.00	.972		.00	.00	.535		.00	.00	.757		.00	.00	.233	
**Variance components**	**Estimate**	**SE**	**Wald Z**	**p**	**Estimate**	**SE**	**Wald Z**	**p**	**Estimate**	**SE**	**Wald Z**	**p**	**Estimate**	**SE**	**Wald Z**	**p**
Residual	1.30	.02	53.29	<.001	1.30	.02	53.29	<.001	1.30	.02	53.29	<.001	1.30	.02	53.29	<.001
Random intercept	.40	.04	1.17	<.001	.40	.04	1.17	<.001	.40	.04	1.17	<.001	.40	.04	1.17	<.001

^a^Grand-mean centered, ^b^Person-mean centered, Reference categories female (sex), weekday (weekend), white (Race/ethnicity), home (location), with others (company)

**Table 4:** Associations between daily physical activity and self-rated cognition analyzed by multilevel modelling (*n* = 2021)

	Memory						Thinking						Sharpness of mind					
	Model 1: Max counts			Model 2: Active time			Model 1: Max counts			Model 2: Active time			Model 1: Max counts			Model 2: Active time		
	B	SE	p	B	SE	p	B	SE	p	B	SE	p	B	SE	p	B	SE	p
**Fixed effects**																		
Intercept	66.03	1.55	<.001	71.84	3.13	<.001	65.08	1.56	<.001	66.56	3.15	<.001	64.61	1.57	<.001	68.38	3.21	<.001
Day	.53	.14	<.001	.56	.16	<.001	.85	.14	<.001	.88	.16	<.001	.71	.14	<.001	.70	.16	<.001
Weekend	2.27	.67	.001	2.25	.68	.001	1.37	.67	.040	1.42	.69	.038	1.77	.68	.010	1.70	.70	.016
Age^a^	.16	.13	.228	.16	.13	.217	.11	.13	.405	.11	.13	.402	.15	.13	.249	.16	.13	.241
Education^a^	.11	.29	.700	.11	.29	.706	.13	.29	.646	.13	.29	.649	.22	.29	.446	.22	.29	.448
Sex	3.86	1.92	.046	3.84	1.92	.047	4.68	1.94	.016	4.68	1.94	.017	5.27	1.94	.007	5.26	1.94	.007
Race/ethnicity	4.83	2.19	.028	4.76	2.19	.030	3.18	2.21	.151	3.17	2.21	.153	4.56	2.22	.041	4.52	2.21	.042
Physical activity^b^	.01	.08	.889	.01	.00	.007	−.03	.08	.663	.00	.00	.810	−.05	.08	.502	.01	.00	.088
**Wear time**				.00	.00	.050				.00	.00	.593				.00	.00	.231
Variance components	**Est.**	**SE**	Wald Z*	**Est.**	**SE**	Wald Z*	**Est.**	**SE**	Wald Z*	**Est.**	**SE**	Wald Z*	**Est.**	**SE**	Wald Z*	**Est.**	**SE**	Wald Z*
Residual	162.47	5.56	29.22	161.92	5.54	29.23	163.29	5.59	29.22	163.39	5.59	29.23	171.50	5.87	29.22	171.40	5.86	29.23
Random intercept	232.98	21.33	1.92	232.60	21.29	1.93	237.68	21.73	1.94	237.46	21.71	1.94	237.18	21.79	1.89	236.68	21.74	1.89

^a^ Grand-mean centered, ^b^Person-mean centered day-level variable. *All Wald Z -tests *p* < 0.001. Reference categories female (sex), weekday (weekend), white (race/ethnicity)

Supplementary materials include models with between-person physical activity variables (Table S2). The within-person associations between physical activity and processing speed remained consistent after accounting for between-person differences in physical activity level. At the between-person level, participants with higher physical activity intensity (maximum counts) had faster processing speed (Table S2). The time lag between the latest physically active minute and cognitive test was not statistically significantly associated with processing speed (Table S4). The within-person association between physical activity and processing speed remained consistent after excluding cases with 15 minutes or longer response delay (Table S6).

Physical activity was unrelated to visual memory at either the between-person or within-person level (Table 3, Table S3, and Table S7). The associations between daily physical activity and self-rated cognition assessed in the evening are in Table 4. At the day-level (RQ2), participants rated their memory better on days when they had more activity minutes than usual. A 1 minute more activity throughout the day was associated with a 0.01 unit increase in self-rated memory (B = 0.01, SE = 0.004, *p* = 0.007). The association between daily activity minutes and memory remained consistent in a model including between-person physical activity (Supplementary Table S5) and after excluding cases with 15 minutes or longer response delay (Supplementary Table S8). There were no between-person associations between physical activity and self-rated cognition (Table S5).

## Discussion

The aim of the present study was to investigate associations between physical activity and cognition in the real-world environment utilizing EMA and accelerometers. Participants had better processing speed (SST) after being more physically active or with higher intensity than their usual. The higher intensity was associated with better processing speed at the between-person level. There were no associations between physical activity and visual memory (DMT). Daily physical activity was mostly unrelated to self-rated cognition except that the participants rated their memory better on days when they had been more physically active than usual.

Both active time and maximum counts were associated with better processing speed. These results are in line with the meta-analysis by Chang et al. [5] suggesting that acute exercise has a positive effect on cognitive tasks that assess information processing, attention, and executive functions in laboratory settings and with studies suggesting that compared to sitting, even standing or light intensity physical activity may improve cognitive performance [34, 35]. There are various possible mechanisms underlying the positive association between physical activity and processing speed. In addition to neurophysiological changes [6, 8], physical activity may lead to higher perceived feelings of alertness and energy [36] while prolonged continuous sitting may lead to higher feelings of fatigue [37]. As the neurophysiological changes may require a higher intensity of physical activity [5], it is possible that in the present study, having any intensity of physical activity is associated with higher feelings of energy and less fatigue, contributing to better processing speed. These same mechanisms may explain why participants rated their memory better than usual on days when they were more physically active. These mechanisms, such as fatigue, feelings of energy, and mood as potential pathways from physical activity to cognition, should be further considered in future studies.

Our results suggest that physical activity performed closer to the cognitive test is more important than physical activity performed in a longer time frame. Previous findings suggest that the largest positive effects are seen 11–20 minutes after exercise and smaller positive effects after 20 minutes delay [5]. In the present study, adding 1 minute of physically active time to the preceding 20 minutes before the cognitive assessment had an over two-fold higher association with improved processing speed compared to adding 1 minute of physical activity to the preceding 60 minutes. The same was the case with physical activity intensity. This finding may be explained by the inclusion of all intensities of physical activity in the present study: lighter physical activities cause less physiological responses and thus their effect on cognition may diminish earlier [5]. This diminishing association may also explain the null associations with self-rated thinking and sharpness of mind: physical activity earlier in the day may not carry throughout the day to the evening assessment. The lack of significant associations between the time lag of physical activity and cognitive outcomes within both the 20- and 60-minute time frames suggests that, in daily life, preceding physical activity is associated with better processing speed regardless of when it occurs within a one-hour time window.

The null findings for visual memory (DMT) are in line with the meta-analysis by Chang et al. [5] suggesting that acute exercise has a positive effect on some cognitive tasks but not on memory. It is also in line with findings that replacing sedentary time with light intensity physical activity is associated with faster task shifting but not working memory [35]. However, there are also opposite findings from a meta-analysis by Lambourne and Tomporowski [4] indicating even stronger effects of acute exercise on memory tasks than processing speed, but their analysis was limited to young adults. It should be noted that the DMT used in the present study, although validated, has also been found not to be associated with other factors that vary across the day [38, 39]. Future studies with other cognitive tests are needed to confirm whether the beneficial association of physical activity is specific to processing speed or occurs with other dimensions of cognitive functioning as well. It should be noted that in the present study, the associations with processing speed were relatively small. For example, every one-minute increase in active time during the last 20 minutes was associated with 3.1 milliseconds faster processing speed. However, when considering the effect size in relation to age, it becomes more meaningful: an increase of 8 minutes in active time is equivalent to the effect of being 1 year younger on processing speed.

The present study was one of the first studies to examine within-person associations between physical activity and cognitive functioning in a real-life environment. This was possible with the novel combination of objective assessments of cognitive performance with mobile phones and physical activity with accelerometers. Participant compliance with both assessment methods was high. There are also some limitations to the study. The community-based sample included relatively inactive (average ~ 4000 steps/day) middle-aged American adults and it would be important to replicate the findings among samples from other age groups, activity-level, and cultural contexts. More research is also needed to assess other cognitive domains in addition to processing speed and visual memory. While the within-subject analyses were based on a large number of assessments, the between-subject findings should be interpreted with caution in the context of the relatively small sample size.

There were some considerations in physical activity assessment that raised questions that could be addressed in future research. For example, it was not possible to separate occupational and leisure time from each other. It would be important to examine whether physical activity in occupational and leisure contexts have similar associations with cognition. While we focused on cognition as an outcome in our analyses, more research is also needed to examine whether this association is bidirectional, i.e., whether people are more physically active when they perform cognitively better. This direction was not examined in the present study as it is likely that occupational physical activity is most dependent on work tasks. The present study focused on activity minutes and maximum intensity in the past 20 and 60 minutes before cognitive assessments. For example, a participant may have accumulated activity minutes consecutively at the beginning or at the end of the time window or nonconsecutively throughout the time window. The maximum intensity captured the peak of physical activity but did not consider the length of the intensity bout. Further studies going deeper on the patterns of physical activity, such as the optimal timing, duration, and intensity of physical activity in daily life for cognitive benefits are recommended. Moreover, participants were allowed to select their preferred wrist (to increase comfort and adherence) for the Actigraph device placement, even though it would have reduced some variability to use either dominant or non-dominant hand for all. This was taken into account in the cut-points and should not affect the within-person associations.

It should also be noted that the data collection was done during the COVID-19 pandemic. It is likely that both participants’ cognition and physical activity patterns may have been affected by the pandemic situation because of the shift to remote work and schooling and the reduction in social contacts [40, 41]. However, data were not collected during the first months of the pandemic (spring 2020), and the data were collected in Florida, where there were fewer restrictions during the data collection period (June 2020–October 2021). It would be important to replicate the results in post-pandemic time.

## Conclusions

In conclusion, this study found that middle-aged adults have better processing speed after they have been physically active. Both spending more time physically active with any intensity compared to sedentary time and doing physical activity with higher intensity have a similar association with processing speed. These associations were stronger in 20-min time window compared to 60-min time window. Results from this study highlight the importance of physical activity throughout the day to boost performance in cognitive tasks requiring processing speed.

### Supplementary Information

**Additional File 1.** information on sample recruitment and representativeness.

**Additional File 2.** STROBE Checklist.

**Additional File 3.** Supplementary tables S1-S8 for the manuscript.

## Data Availability

The datasets generated and/or analysed during the current study are not publicly available because the restricted geographical location from which participants were recruited increases risk of re-identification but are available from the corresponding author on reasonable request.

## Abbreviations

EMA: Ecological momentary assessment
RQ: Research question
CHAP: Couples Healthy Aging Project
SST: Symbol Search Task
DMT: Dot Memory test
VMC: Vector magnitude counts
CPM: Count per minute
ICC: Intraclass correlation coefficient
VC: Variance components
REML: Restricted maximum likelihood
