# Supplementary material for: Physical activity and cognitive function: moment-to-moment and day-to-day associations
Source: Int J Behav Nutr Phys Act. 2023 Nov 22;20:137. doi: 10.1186/s12966-023-01536-9 (PMC10666351; doi:10.1186/s12966-023-01536-9)
Supplement: Supplementary file 1 — Additional File 1. information on sample recruitment and representativeness. [file 12966_2023_1536_MOESM1_ESM.docx]

**Physical Activity and Cognitive Function: Moment-to-Moment and Day-to-Day Associations**

**Additional File 1**

**Recruitment**

Participants were recruited through social media advertisements, community events, and snowball sampling. The inclusion criteria were 1) both members of the couple were aged 40 to 70 years, 2) in a committed relationship for at least one year and cohabitating, and 3) both members of the couple were cognitively unimpaired and willing to enroll in the study.

**Representativeness of the sample**

All participants lived locally in Tallahassee, FL. Because of the age and relationship inclusion criteria for the current study, it is difficult to assess the representativeness of the sample compared to the population of Tallahassee. For example, the percentage of the sample who was of color was 24.7%. The current Census estimates for the percentage of people of color in Tallahassee in 2020 was 46.7%, but it is likely to be lower than that for our target age group because of demographic shifts (i.e., older populations of Tallahassee have a higher percentage of white people than younger populations). Likewise, the average education in Tallahassee is a bachelor’s degree, which is the same in our sample, but is likely to be an overestimate for our target age (i.e., younger populations are more likely to have a bachelor’s degree). Age is not representative because the focus on middle-aged adults was central to the research question of the parent study. The distribution of sex is roughly similar to the population of Tallahassee (52.8% in Tallahassee versus 54.6% in the current study).

Of the 308 participants recruited, 98% (n = 303) had valid data from the EMA portion of the study (n=5 data were lost due to technical problems with the phone) and 96% (n = 296) wore the accelerometer. The present study includes participants who had information on both EMA and physical activity from at least one day (n = 291). The comparison between the analytic sample (n = 291) and those excluded from the present study (n = 17) is presented in Table 1. The analytic sample did not differ from the rest of the recruited participants in terms of sociodemographic factors, health status, or cognitive functioning.

Table 1. The comparison between the analytic sample (n = 291) and those excluded from the present study (n = 17)

|  | Analytic sample (n = 291) | Excluded from the analysis  (n = 17) | Difference test |
| --- | --- | --- | --- |
|  | M ± SD | M ± SD | t (df), p |
| Age, y | 51.8 ± 7.4 | 50.1 ± 5.1 | -.97 (306), .334 |
| Education, y | 16.6 ± 3.3 | 16.6 ± 2.6 | -.02 (306), .983 |
| Body mass index | 30.8 ± 6.6 | 30.6 ± 5.6 | -.17 (302), .863 |
| Number of diseases^a^ | 0.1 ± 0.6 | 0 ± 0 | -.70 (304), .498 |
| Self-rated health^b^ | 3.4 ± 0.9 | 3.3 ± 0.7 | -.25 (303), .807 |
| Verbal fluency^c^ | 23.9 ± 6.0 | 25.2 ± 5.1 | .84 (301), .399 |
| Cognitive status summary score^d^ | 17.4 ± 3.0 | 18.3 ± 3.8 | 1.1 (302), .259 |
| Self-rated memory^e^ | 3.2 ± 0.8 | 3.2 ± 1.0 | .02 (301), .983 |
|  | N (%) | N (%) | X^2^ (df), p |
| Sex |  |  | 1.17 (1), .279 |
| Male | 132 (45.4) | 10 (58.8) |  |
| Female | 159 (54.6) | 7 (41.2) |  |
| Race/ethnicity |  |  | .09 (1), .764 |
| White | 215 (73.9) | 12 (70.6) |  |
| Person of color | 76 (24.7) | 5 (29.4) |  |

^a^ Sum of diagnosed diseases (0-7) including hypertension, diabetes(any), cancer (exc. skin), heart condition, stroke, arthritis, and lung disease

^b^ In general, would you say your health is...? (Scale: 1=poor – 5=excellent)

^c^ A total number of animals named by a participant within 60 s (Lezak 2004)

^d^ TICS: Sum of scores from immediate and delayed word recall, count backwards, and serial 7 (Crimmins et al. 2011)

^e^ How would you rate your memory at the present time? (Scale: 1=poor – 5=excellent)

**Missing values**

**EMA-level analysis:** A total of 290 participants provided information on cognitive tests and physical activity at least one assessment point, resulting in a maximum number of 6960 cases (3 assessments per day * 8 days * 290 participants = 6960). Most missing values are due to completely missing EMA assessments in which the participants did not start the assessment at all (9% of cases, n = 627). The number of available information is presented in Table 2 for main variables. The data were analyzed with restricted maximum likelihood (REML) estimation using all available data to estimate the model parameters.

Table 2. Information available for the EMA-level analysis

| Variable | Cases | % of cases (6960) | % of started beeps (6333) |
| --- | --- | --- | --- |
| (Symbol Search Task | 6256 | 89.9 | 98.8 |
| Dot Memory | 6078 | 87.3 | 96.0 |
| Accelerometer information 20 min before EMA | 6298 | 90.5 | 99.4 |
| Accelerometer information 60 min before EMA | 6298 | 90.5 | 99.4 |
| Symbol Search Task & Accelerometer | 6221 | 89.4 | 98.2 |
| Dot Memory & Accelerometer | 6045 | 86.9 | 95.5 |

Information on other temporal covariates (EMA day and session number, weekend, and company) was available for all cases having Symbol Search Task or Dot Memory information. The information on location was missing for 2 and 3 cases having Symbol Search Task or Dot Memory information, respectively. Information on all between-person covariates (age, education, sex, and race/ethnicity) was available from all participants (n = 290).

**Day-level analysis:** A total of 291 participants had information on self-rated cognition and physical activity from at least one day leading to a maximum number of 2328 cases (1 assessment per day * 8 days * 291 participants = 2328). Most missing values are due to completely missing evening assessments in which the participants did not start the assessment at all (7% of cases, n = 171).

The number of available information is presented in Table 3 for main variables. The data were analyzed with restricted maximum likelihood (REML) estimation using all available data to estimate the model parameters.

Table 3. Information available for the day-level analysis

| Variable | Cases | % of cases (2328) | % of started beeps (2157) |
| --- | --- | --- | --- |
| Memory | 2145 | 92.1 | 99.4 |
| Thinking | 2144 | 92.1 | 99.4 |
| Sharpness of mind | 2145 | 92.1 | 99.4 |
| Accelerometer information from the day | 2183 | 93.8 | 93.8 |
| Memory/thinking/sharpness & Accelerometer | 2012 | 86.4 | 93.3 |

Information on temporal covariates (EMA day and weekend) was available for all cases having self-rated cognition information. Information on all between-person covariates (age, education, sex, and race/ethnicity) was available from all participants (n = 291).

**References**

Lezak, M. D. (2004). Neuropsychological assessment (4th ed.). New York: Oxford University Press.

Crimmins EM, Kim JK, Langa KM, Weir DR. (2011). Assessment of cognition using surveys and neuropsychological assessment: the Health and Retirement Study and the Aging, Demographics, and Memory Study. J Gerontol B Psychol Sci Soc Sci. 66 Suppl 1:i162-71. doi:10.1093/geronb/gbr048
