# Supplementary material for: Physical activity and cognitive function: moment-to-moment and day-to-day associations
Source: Int J Behav Nutr Phys Act. 2023 Nov 22;20:137. doi: 10.1186/s12966-023-01536-9 (PMC10666351; doi:10.1186/s12966-023-01536-9)
Supplement: Supplementary file 3 — Additional File 3. Supplementary tables S1-S8 for the manuscript. [file 12966_2023_1536_MOESM3_ESM.docx]

**Physical Activity and Cognitive Function: Moment-to-Moment and Day-to-Day Associations**

**Supplementary materials**

**Table S1. Within-person day-level (above the diagonal, n=1954) and between-person (below the diagonal (N=291) correlations between study variables**

|  | 1 | 2 | 3 | 4 | 5 | 6 | 7 | 8 |
| --- | --- | --- | --- | --- | --- | --- | --- | --- |
| 1. Processing speed |  | .02 | -.02 | -.02 | -.02 | .00 | .00 |  |
| 2. Visual memory | .25*** |  |  | -.02 | .00 | .00 | .01 |  |
| 3. Self-rated memory | .09 | .00 |  | .70*** | .69*** | .16*** | .00 |  |
| 4. Self-rated thinking | .06 | .02 | .95*** |  | .65*** | .01 | .00 |  |
| 5. Self-rated mind-sharpness | .11 | -.01 | .96*** | .96*** |  | .08* | .00 |  |
| 6. Active time | -.13* | .02 | .01 | .03 | .02 |  | .00 |  |
| 7. Maximum counts | -.33*** | -.07 | .03 | .02 | .01 | .46*** |  |  |
| 8. Age | .40*** | .22*** | .08 | .07 | .08 | -.16** | -.34*** |  |
| 9. Education | -.10 | .15* | .01 | .01 | .02 | -.03 | .13* | -.14* |

*p<.05, **p<.01, ***p<.001

**Table S2. Associations between physical activity and processing speed analyzed by multilevel modelling. Models including between-person physical activity.**

|  | **Model: Max Counts 60 min** | | | | **Model: Max Counts 20 min** | | | | **Model: Active time 60 min** | | | | | **Model: Active time 20 min** | | | |
| --- | --- | --- | --- | --- | --- | --- | --- | --- | --- | --- | --- | --- | --- | --- | --- | --- | --- |
|  | **B** | **SE** | **p** |  | **B** | **SE** | **p** |  | **B** | **SE** | **p** |  | **B** | | **SE** | **p** |  |
| **Fixed effects** |  |  |  |  |  |  |  |  |  |  |  |  |  | |  |  |  |
| Intercept | 1859.19 | 53.46 | <.001 |  | 1853.98 | 53.29 | <.001 |  | 1829.48 | 54.46 | <.001 |  | 1831.21 | | 54.45 | <.001 |  |
| EMA day | -27.48 | 1.78 | <.001 |  | -27.43 | 1.78 | <.001 |  | -27.51 | 1.78 | <.001 |  | -27.45 | | 1.78 | <.001 |  |
| EMA session | 4.18 | 4.87 | .391 |  | 3.92 | 4.87 | .421 |  | 5.10 | 4.88 | .297 |  | 4.63 | | 4.87 | .342 |  |
| Weekend | -8.92 | 9.43 | .344 |  | -9.45 | 9.43 | .316 |  | -8.71 | 9.43 | .356 |  | -9.05 | | 9.43 | .337 |  |
| Company | -23.55 | 9.05 | .009 |  | -24.19 | 9.06 | .008 |  | -24.49 | 9.06 | .007 |  | -24.83 | | 9.07 | .006 |  |
| Location | -22.11 | 9.30 | .017 |  | -24.52 | 9.30 | .008 |  | -2.83 | 9.31 | .025 |  | -23.18 | | 9.29 | .013 |  |
| Age^a^ | 19.12 | 3.32 | <.001 |  | 19.09 | 3.31 | <.001 |  | 22.91 | 3.25 | <.001 |  | 22.90 | | 3.25 | <.001 |  |
| Education^a^ | -2.05 | 6.88 | .766 |  | -2.01 | 6.87 | .771 |  | -2.09 | 7.06 | .768 |  | -2.09 | | 7.06 | .768 |  |
| Sex | 107.27 | 46.16 | .021 |  | 107.49 | 46.12 | .020 |  | 113.21 | 47.91 | .019 |  | 113.31 | | 47.91 | .019 |  |
| Race/ethnicity | -179.68 | 52.65 | .001 |  | -179.61 | 52.61 | .001 |  | -178.54 | 53.99 | .001 |  | -178.45 | | 53.99 | .001 |  |
| Physical activity^b^ | -.19 | .05 | <.001 |  | -.49 | .13 | <.001 |  | -1.26 | .30 | <.001 |  | -3.11 | | .70 | <.001 |  |
| Physical activity^a^ | -3.00 | 7.45 | <.001 |  | -3.75 | 7.44 | <.001 |  | -.38 | .23 | .108 |  | -.38 | | .23 | .109 |  |
| **Variance components** | **Estimate** | **SE** | **Wald Z** | **p** | **Estimate** | **SE** | **Wald Z** | **p** | **Estimate** | **SE** | **Wald Z** | **p** | **Estimate** | | **SE** | **Wald Z** | **p** |
| Residual | 95532.88 | 1764.44 | 54.14 | <.001 | 9553.02 | 1764.39 | 54.14 | <.001 | 95489.30 | 1763.64 | 54.14 | <.001 | 95464.68 | | 1763.18 | 54.14 | <.001 |
| Random intercept | 141572.9 | 12115.44 | 11.69 | <.001 | 141352.37 | 12097.18 | 11.68 | <.001 | 149121.81 | 12741.46 | 11.70 | <.001 | 149121.69 | | 12741.35 | 11.70 | <.001 |

^a^Grand-mean centered, ^b^Person-mean centered, Reference categories female (sex), weekday (weekend), white (Race/ethnicity), location (home), company (with others).

**Table S3. Associations between physical activity and visual memory analyzed by multilevel modelling. Models including between-person physical activity.**

|  | **Model: Max Counts 60 min** | | | | **Model: Max Counts 20 min** | | | | **Model: Active time 60 min** | | | | **Model: Active time 20 min** | | | |
| --- | --- | --- | --- | --- | --- | --- | --- | --- | --- | --- | --- | --- | --- | --- | --- | --- |
|  | **B** | **SE** | **p** |  | **B** | **SE** | **p** |  | **B** | **SE** | **p** |  | **B** | **SE** | **p** |  |
| **Fixed effects** |  |  |  |  |  |  |  |  |  |  |  |  |  |  |  |  |
| Intercept | 1.94 | .11 | <.001 |  | 1.95 | .11 | <.001 |  | 1.94 | .11 | <.001 |  | 1.94 | .11 | <.001 |  |
| EMA day | -.05 | .01 | <.001 |  | -.05 | .01 | <.001 |  | -.05 | .01 | <.001 |  | -.05 | .01 | <.001 |  |
| EMA session | .08 | .02 | <.001 |  | .08 | .02 | <.001 |  | .08 | .02 | <.001 |  | .08 | .02 | <.001 |  |
| Weekend | .00 | .04 | .941 |  | .00 | .04 | .950 |  | .00 | .04 | .951 |  | .00 | .04 | .965 |  |
| Company | -.05 | .03 | .129 |  | -.05 | .03 | .119 |  | -.05 | .03 | .125 |  | -.05 | .03 | .107 |  |
| Location | .03 | .03 | .424 |  | .03 | .03 | .432 |  | .03 | .03 | .414 |  | .03 | .03 | .424 |  |
| Age^a^ | .02 | .01 | <.001 |  | .02 | .01 | <.001 |  | .03 | .01 | <.001 |  | .03 | .01 | <.001 |  |
| Education^a^ | -.03 | .01 | .015 |  | -.03 | .01 | .015 |  | -.03 | .01 | .016 |  | -.03 | .01 | .016 |  |
| Sex | -.35 | .08 | <.001 |  | -.35 | .08 | <.001 |  | -.35 | .08 | <.001 |  | -.35 | .08 | <.001 |  |
| Race/ethnicity | .34 | .09 | <.001 |  | .33 | .09 | <.001 |  | .33 | .09 | <.001 |  | .33 | .09 | <.001 |  |
| Physical activity^b^ | .00 | .00 | .993 |  | .00 | .00 | .541 |  | .00 | .00 | .757 |  | .00 | .00 | .233 |  |
| Physical activity^a^ | .00 | .01 | .765 |  | .00 | .01 | .780 |  | .00 | .00 | .940 |  | .00 | .00 | .943 |  |
| **Variance components** | **Estimate** | **SE** | **Wald Z** | **p** | **Estimate** | **SE** | **Wald Z** | **p** | **Estimate** | **SE** | **Wald Z** | **p** | **Estimate** | **SE** | **Wald Z** | **p** |
| Residual | 1.30 | .02 | 53.29 | <.001 | 1.30 | .02 | 53.29 | <.001 | 1.30 | .02 | 53.29 | <.001 | 1.30 | .02 | 53.29 | <.001 |
| Random intercept | .40 | .04 | 1.16 | <.001 | .40 | .04 | 1.16 | <.001 | .40 | .04 | 1.16 | <.001 | .40 | .04 | 1.16 | <.001 |

^a^Grand-mean centered, ^b^Person-mean centered, Reference categories female (sex), weekday (weekend), white (Race/ethnicity), location (home), company (with others).

**Table S4. Associations between physical activity and cognitive performance analyzed by multilevel modelling. Models including the time lag between the last physically active minute and cognitive test.**

|  | **Processing speed** | | | | | | | | **Visual memory** | | | | | | | | |
| --- | --- | --- | --- | --- | --- | --- | --- | --- | --- | --- | --- | --- | --- | --- | --- | --- | --- |
|  | **Model: Active time 60 min** | | | | **Model: Active time 20 min** | | | | **Model: Active time 60 min** | | | | | **Model: Active time 20 min** | | | |
|  | **B** | **SE** | **p** |  | **B** | **SE** | **p** |  | **B** | **SE** | **p** |  | **B** | | **SE** | **p** |  |
| **Fixed effects** |  |  |  |  |  |  |  |  |  |  |  |  |  | |  |  |  |
| Intercept | 1818.89 | 55.12 | <.001 |  | 1816.05 | 55.75 | <.001 |  | 1.97 | .11 | <.001 |  | 1.93 | | .12 | <.001 |  |
| EMA day | -26.37 | 1.81 | <.001 |  | -25.65 | 1.99 | <.001 |  | -.05 | .01 | <.001 |  | -.04 | | .01 | <.001 |  |
| EMA session | 6.24 | 4.97 | .056 |  | 10.56 | 5.53 | .056 |  | .08 | .02 | <.001 |  | .08 | | .02 | <.001 |  |
| Weekend | -8.88 | 9.64 | .144 |  | -15.74 | 10.77 | .144 |  | -.01 | .04 | .790 |  | .01 | | .04 | .819 |  |
| Company | -21.63 | 9.21 | .081 |  | -17.81 | 10.20 | .081 |  | -.05 | .03 | .128 |  | -.05 | | .04 | .190 |  |
| Location | -19.01 | 9.50 | .096 |  | -17.38 | 10.45 | .096 |  | .02 | .04 | .571 |  | -.00 | | .04 | .918 |  |
| Age^a^ | 23.45 | 3.24 | <.001 |  | 23.29 | 3.23 | <.001 |  | .03 | .01 | <.001 |  | .03 | | .01 | <.001 |  |
| Education^a^ | -2.45 | 7.11 | .824 |  | -1.58 | 7.07 | .824 |  | -.03 | .01 | .012 |  | -.03 | | .01 | .017 |  |
| Sex | 126.64 | 47.52 | .008 |  | 126.96 | 47.30 | .008 |  | -.35 | .08 | <.001 |  | -.36 | | .08 | <.001 |  |
| Race/ethnicity | -179.21 | 54.46 | <.001 |  | -181.37 | 54.25 | <.001 |  | .33 | .10 | <.001 |  | .33 | | .10 | <.001 |  |
| Physical activity^b^ | -.95 | .32 | .003 |  | -2.41 | .90 | .008 |  | .00 | .00 | .695 |  | -.00 | | .00 | .550 |  |
| Time lag^c^ | -.43 | .38 | .261 |  | -1.58 | .95 | .097 |  | -.00 | .00 | .667 |  | .00 | | .00 | .397 |  |
| **Variance components** | **Estimate** | **SE** | **Wald Z** | **p** | **Estimate** | **SE** | **Wald Z** | **p** | **Estimate** | **SE** | **Wald Z** | **p** | **Estimate** | | **SE** | **Wald Z** | **p** |
| Residual | 92672.85 | 1763.35 | 52.56 | <.001 | 93568.44 | 1957.42 | 47.80 | <.001 | 1.30 | .03 | 51.70 | <.001 | 1.30 | | .03 | 47.03 | <.001 |
| Random intercept | 151400.73 | 12971.84 | 11.67 | <.001 | 148820.62 | 12852.87 | 11.58 | <.001 | .40 | .04 | 10.08 | <.001 | .40 | | .04 | 9.75 | <.001 |

^a^Grand-mean centered, ^b^Person-mean centered, ^c^Time lag between the last activity minute and cognitive assessment. Reference categories female (sex), weekday (weekend), white (Race/ethnicity), location (home), company (with others).

**Table S5. Associations between daily physical activity and self-rated cognition analyzed by multilevel modelling. Models including between-person physical activity.**

|  | **Memory** | | | | | | **Thinking** | | | | | | **Sharpness of mind** | | | | | |
| --- | --- | --- | --- | --- | --- | --- | --- | --- | --- | --- | --- | --- | --- | --- | --- | --- | --- | --- |
|  | **Model 1: Max counts** | | | **Model 2: Active time** | | | **Model 1: Max counts** | | | **Model 2: Active time** | | | **Model 1: Max counts** | | | **Model 2: Active time** | | |
|  | **B** | **SE** | **p** | **B** | **SE** | **p** | **B** | **SE** | **p** | **B** | **SE** | **p** | **B** | **SE** | **p** | **B** | **SE** | **p** |
| **Fixed effects** |  |  |  |  |  |  |  |  |  |  |  |  |  |  |  |  |  |  |
| Intercept | 65.94 | 1.55 | <.001 | 71.72 | 3.13 | <.001 | 65.01 | 1.56 | <.001 | 66.40 | 3.15 | <.001 | 64.54 | 1.57 | <.001 | 68.24 | 3.21 | <.001 |
| Day | .53 | .14 | <.001 | .56 | .16 | <.001 | .85 | .14 | <.001 | .88 | .16 | <.001 | .71 | .14 | <.001 | .70 | .16 | <.001 |
| Weekend | 2.27 | .67 | .001 | 2.25 | .68 | .001 | 1.37 | .67 | .040 | 1.42 | .69 | .038 | 1.77 | .68 | .010 | 1.70 | .70 | .015 |
| Age^a^ | .21 | .14 | .130 | .18 | .13 | .168 | .16 | .14 | .264 | .14 | .13 | .299 | .20 | .14 | .157 | .18 | .13 | .177 |
| Education^a^ | .12 | .29 | .691 | .12 | .29 | .668 | .14 | .29 | .639 | .15 | .29 | .601 | .23 | .29 | .440 | .24 | .29 | .412 |
| Sex | 4.06 | 1.93 | .036 | 4.23 | 1.95 | .031 | 4.86 | 1.95 | .013 | 5.20 | 1.97 | .009 | 5.45 | 1.95 | .006 | 5.74 | 1.97 | .004 |
| Race/ethnicity | -4.82 | 2.19 | .029 | -4.77 | 2.19 | .030 | -3.17 | 2.21 | .153 | -3.17 | 2.21 | .152 | -4.55 | 2.22 | .041 | -4.52 | 2.21 | .042 |
| Physical activity^b^ | .01 | .08 | .891 | .01 | .00 | .007 | -.03 | .08 | .662 | .00 | .00 | .798 | -.05 | .08 | .501 | .01 | .00 | .085 |
| Physical activity^a^ | .36 | .31 | .248 | .01 | .01 | .271 | .32 | .31 | .311 | .01 | .01 | .148 | .32 | .31 | .317 | .01 | .01 | .187 |
| Wear time |  |  |  | .00 | .00 | .048 |  |  |  | .00 | .00 | .581 |  |  |  | .00 | .00 | .225 |
| **Variance components** | **Est.** | **SE** | **Wald Z*** | **Est.** | **SE** | **Wald Z*** | **Est.** | **SE** | **Wald Z*** | **Est.** | **SE** | **Wald Z*** | **Est.** | **SE** | **Wald Z*** | **Est.** | **SE** | **Wald Z*** |
| Residual | 162.47 | 5.56 | 29.22 | 161.91 | 5.54 | 29.23 | 163.28 | 5.59 | 29.22 | 163.37 | 5.59 | 29.23 | 171.49 | 5.87 | 29.22 | 171.38 | 5.86 | 29.23 |
| Random intercept | 232.70 | 21.27 | 1.94 | 232.53 | 21.24 | 1.95 | 237.67 | 21.69 | 1.96 | 236.61 | 21.60 | 1.95 | 237.20 | 21.75 | 1.91 | 236.16 | 21.66 | 1.90 |

^a^ Grand-mean centered, ^b^Person-mean centered day-level variable. *All Wald Z -tests p<0.001. Reference categories female (sex), weekday (weekend), white (race/ethnicity).

**Table S6. Associations between physical activity and processing speed analyzed by multilevel modelling. Models including between-person physical activity. Models including only cases with less than 15 minutes response delay (n = 4556).**

|  | **Model: Max Counts 60 min** | | | | **Model: Max Counts 20 min** | | | | **Model: Active time 60 min** | | | | **Model: Active time 20 min** | | | |
| --- | --- | --- | --- | --- | --- | --- | --- | --- | --- | --- | --- | --- | --- | --- | --- | --- |
|  | **B** | **SE** | **p** |  | **B** | **SE** | **p** |  | **B** | **SE** | **p** |  | **B** | **SE** | **p** |  |
| **Fixed effects** |  |  |  |  |  |  |  |  |  |  |  |  |  |  |  |  |
| Intercept | 1837.77 | 56.98 | <.001 |  | 1825.84 | 56.87 | <.001 |  | 1801.82 | 56.67 | <.001 |  | 1804.34 | 56.71 | <.001 |  |
| EMA day | -27.33 | 2.03 | <.001 |  | -27.49 | 2.03 | <.001 |  | -27.40 | 2.03 | <.001 |  | -27.30 | 2.03 | <.001 |  |
| EMA session | 3.39 | 5.63 | .547 |  | 2.71 | 5.63 | .630 |  | 4.35 | 5.64 | .440 |  | 3.34 | 5.63 | .553 |  |
| Weekend | -8.88 | 10.85 | .413 |  | -10.32 | 10.85 | .342 |  | -8.60 | 10.84 | .428 |  | -9.78 | 10.85 | .367 |  |
| Company | -16.45 | 10.34 | .037 |  | -16.71 | 10.38 | .107 |  | -17.66 | 10.38 | .089 |  | -17.12 | 10.39 | .099 |  |
| Location | -22.58 | 10.83 | .037 |  | -26.43 | 10.83 | .015 |  | -21.58 | 10.84 | .047 |  | -25.30 | 10.82 | .019 |  |
| Age^a^ | 22.08 | 3.29 | <.001 |  | 22.18 | 3.30 | <.001 |  | 22.34 | 3.30 | <.001 |  | 22.29 | 3.31 | <.001 |  |
| Education^a^ | -2.14 | 7.22 | .767 |  | -2.08 | 7.24 | .774 |  | -1.98 | 7.24 | .785 |  | -2.04 | 7.25 | .779 |  |
| Sex | 135.51 | 48.54 | .006 |  | 137.34 | 48.63 | .005 |  | 139.64 | 48.65 | .004 |  | 140.21 | 48.69 | .004 |  |
| Race/ethnicity | 155.31 | 55.69 | .006 |  | 155.19 | 55.80 | .006 |  | 157.65 | 55.83 | .005 |  | 156.94 | 55.88 | .005 |  |
| Physical activity^b^ | -.28 | .06 | <.001 |  | -.60 | .15 | <.001 |  | -1.26 | .35 | <.001 |  | -3.20 | .81 | <.001 |  |
| **Variance components** | **Estimate** | **SE** | **Wald Z** | **p** | **Estimate** | **SE** | **Wald Z** | **p** | **Estimate** | **SE** | **Wald Z** | **p** | **Estimate** | **SE** | **Wald Z** | **p** |
| Residual | 91399.90 | 1981.50 | 46.13 | <.001 | 91485.18 | 1983.35 | 46.13 | <.001 | 91348.58 | 1980.39 | 46.13 | <.001 | 95464.68 | 1763.18 | 46.13 | <.001 |
| Random intercept | 152036.38 | 13335.39 | 11.40 | <.001 | 152623.76 | 13385.42 | 11.40 | <.001 | 152821.50 | 13401.49 | 11.40 | <.001 | 149121.69 | 12741.35 | 11.40 | <.001 |

^a^Grand-mean centered, ^b^Person-mean centered, Reference categories female (sex), weekday (weekend), white (Race/ethnicity), home (location), with others (company).

**Table S7. Associations between physical activity and visual memory analyzed by multilevel modelling. Models including only cases with less than 15 minutes response delay (n = 4432).**

|  | **Model: Max Counts 60 min** | | | | **Model: Max Counts 20 min** | | | | **Model: Active time 60 min** | | | | **Model: Active time 20 min** | | | |
| --- | --- | --- | --- | --- | --- | --- | --- | --- | --- | --- | --- | --- | --- | --- | --- | --- |
|  | **B** | **SE** | **p** |  | **B** | **SE** | **p** |  | **B** | **SE** | **p** |  | **B** | **SE** | **p** |  |
| **Fixed effects** |  |  |  |  |  |  |  |  |  |  |  |  |  |  |  |  |
| Intercept | 1.89 | .12 | <.001 |  | 1.89 | .12 | <.001 |  | 1.89 | .12 | <.001 |  | 1.87 | .11 | <.001 |  |
| EMA day | -.05 | .01 | <.001 |  | -.05 | .01 | <.001 |  | -.05 | .01 | <.001 |  | -.05 | .01 | <.001 |  |
| EMA session | .11 | .02 | <.001 |  | .11 | .02 | <.001 |  | .11 | .02 | <.001 |  | .11 | .02 | <.001 |  |
| Weekend | .01 | .04 | .899 |  | .01 | .04 | .901 |  | .01 | .04 | .889 |  | .01 | .04 | .900 |  |
| Company | -.03 | .04 | .487 |  | -.03 | .04 | .480 |  | -.03 | .04 | .464 |  | -.03 | .04 | .445 |  |
| Location | .03 | .04 | .480 |  | .03 | .04 | .484 |  | .03 | .04 | .461 |  | .03 | .04 | .485 |  |
| Age^a^ | .03 | .01 | <.001 |  | .03 | .01 | <.001 |  | .03 | .01 | <.001 |  | .03 | .01 | <.001 |  |
| Education^a^ | -.03 | .01 | .018 |  | -.03 | .01 | .018 |  | -.03 | .01 | .018 |  | -.03 | .01 | .018 |  |
| Sex | -.39 | .08 | <.001 |  | -.39 | .08 | <.001 |  | -.39 | .08 | <.001 |  | -.39 | .08 | <.001 |  |
| Race/ethnicity | .33 | .10 | <.001 |  | .33 | .10 | <.001 |  | .33 | .10 | <.001 |  | .33 | .10 | <.001 |  |
| Physical activity^b^ | -.00 | .00 | .969 |  | -.00 | .00 | .839 |  | -.00 | .00 | .611 |  | -.00 | .00 | .395 |  |
| **Variance components** | **Estimate** | **SE** | **Wald Z** | **p** | **Estimate** | **SE** | **Wald Z** | **p** | **Estimate** | **SE** | **Wald Z** | **p** | **Estimate** | **SE** | **Wald Z** | **p** |
| Residual | 1.29 | .03 | 45.48 | <.001 | 1.29 | .03 | 45.48 | <.001 | 1.29 | .03 | 45.48 | <.001 | 1.29 | .03 | 45.48 | <.001 |
| Random intercept | .37 | .04 | 9.51 | <.001 | .37 | .04 | 9.51 | <.001 | .37 | .04 | 9.51 | <.001 | .37 | .04 | 9.51 | <.001 |

^a^Grand-mean centered, ^b^Person-mean centered, Reference categories female (sex), weekday (weekend), white (Race/ethnicity), home (location), with others (company).

**Table S8.** **Associations between daily physical activity and self-rated cognition analyzed by multilevel modelling. Models including only cases with less than 15 minutes response delay (n = 1716).**

|  | **Memory** | | | | | | **Thinking** | | | | | | **Sharpness of mind** | | | | | |
| --- | --- | --- | --- | --- | --- | --- | --- | --- | --- | --- | --- | --- | --- | --- | --- | --- | --- | --- |
|  | **Model 1: Max counts** | | | **Model 2: Active time** | | | **Model 1: Max counts** | | | **Model 2: Active time** | | | **Model 1: Max counts** | | | **Model 2: Active time** | | |
|  | **B** | **SE** | **p** | **B** | **SE** | **p** | **B** | **SE** | **p** | **B** | **SE** | **p** | **B** | **SE** | **p** | **B** | **SE** | **p** |
| **Fixed effects** |  |  |  |  |  |  |  |  |  |  |  |  |  |  |  |  |  |  |
| Intercept | 71.11 | 2.37 | <.001 | 79.64 | 3.87 | <.001 | 69.44 | 2.37 | <.001 | 73.44 | 3.88 | <.001 | 69.14 | 2.40 | <.001 | 75.97 | 3.21 | <.001 |
| Day | .44 | .16 | .005 | .46 | .18 | <.001 | .73 | .16 | <.001 | .74 | .18 | <.001 | .65 | .16 | <.001 | .62 | .16 | <.001 |
| Weekend | 2.23 | .75 | .003 | 2.20 | .76 | .001 | .94 | .75 | .208 | .95 | .77 | .216 | 2.02 | .77 | .009 | 1.91 | .70 | .015 |
| Age^a^ | .20 | .14 | .151 | .21 | .14 | .217 | .16 | .14 | .257 | .16 | .14 | .246 | .22 | .14 | .118 | .23 | .13 | .106 |
| Education^a^ | .14 | .30 | .644 | .15 | .31 | .706 | .19 | .30 | .526 | .20 | .30 | .520 | .24 | .31 | .440 | .25 | .29 | .429 |
| Sex | 4.08 | 2.01 | .044 | 4.04 | 2.02 | .047 | 4.50 | 2.01 | .026 | 4.48 | 2.01 | .027 | 5.50 | 2.04 | .007 | 5.46 | 1.94 | .008 |
| Race/ethnicity | 4.94 | 2.30 | .033 | 4.89 | 2.31 | .030 | 3.87 | 2.30 | .094 | 3.85 | 2.30 | .096 | 4.90 | 2.33 | .036 | 4.86 | 2.21 | .038 |
| Physical activity^b^ | .02 | .08 | .817 | .02 | .00 | .007 | -.01 | .09 | .915 | .01 | .00 | .127 | -.03 | .09 | .751 | .01 | .00 | .002 |
| Wear time |  |  |  | -.01 | .00 | .011 |  |  |  | -.00 | .00 | .231 |  |  |  | -.00 | .00 | .056 |
| **Variance components** | **Est.** | **SE** | **Wald Z*** | **Est.** | **SE** | **Wald Z*** | **Est.** | **SE** | **Wald Z*** | **Est.** | **SE** | **Wald Z*** | **Est.** | **SE** | **Wald Z*** | **Est.** | **SE** | **Wald Z*** |
| Residual | 158.17 | 6.14 | 25.76 | 156.54 | 6.07 | 25.77 | 159.39 | 6.19 | 25.76 | 159.19 | 6.17 | 25.77 | 167.73 | 6.51 | 25.76 | 166.61 | 6.47 | 25.77 |
| Random intercept | 242.10 | 22.96 | 10.54 | 243.74 | 23.07 | 10.56 | 241.56 | 22.94 | 10.53 | 241.81 | 22.95 | 10.54 | 247.31 | 23.56 | 10.50 | 248.23 | 23.16 | 10.51 |

^a^ Grand-mean centered, ^b^Person-mean centered day-level variable. *All Wald Z -tests p<0.001. Reference categories female (sex), weekday (weekend), white (race/ethnicity).
